# UPLC/Q-TOF MS-Based Urine Metabonomics Study to Identify Diffuse Axonal Injury Biomarkers in Rat

**DOI:** 10.1155/2022/2579489

**Published:** 2022-09-21

**Authors:** Peng Zhang, Sheng Wang, Meiqing Liu, Wenhui Li, Leilei Li, Shisheng Zhu, Qianyun Nie, Qifu Li

**Affiliations:** ^1^Department of Neurology, The First Affiliated Hospital of Hainan Medical University, Haikou 570102, China; ^2^Department of Forensic Medicine, Hainan Medical University, Haikou 571199, China; ^3^Faculty of Medical Technology, Chongqing Medical and Pharmaceutical College, Chongqing 401331, China; ^4^School of Forensic Medicine, Kunming Medical University, Kunming 650500, China

## Abstract

Diffuse axonal injury (DAI) represents a frequent traumatic brain injury (TBI) type, significantly contributing to the dismal neurological prognosis and high mortality in TBI patients. The increase in mortality can be associated with delayed and nonspecific initial symptoms in DAI patients. Additionally, the existing approaches for diagnosis and monitoring are either low sensitivity or high cost. Therefore, novel, reliable, and objective diagnostic markers should be developed to diagnose and monitor DAI prognosis. Urine is an optimal sample to detect biomarkers for DAI noninvasively. Therefore, the DAI rat model was established in this work. Meanwhile, the ultraperformance liquid chromatography quadrupole-time-of-flight hybrid mass spectrometry- (UPLC/Q-TOF MS-) untargeted metabolomics approach was utilized to identify the features of urine metabolomics to diagnose DAI. This work included 57 metabolites with significant alterations and 21 abnormal metabolic pathways from the injury groups. Three metabolites, viz., urea, butyric acid, and taurine, were identified as possible biomarkers to diagnose DAI based on the great fold changes (FCs) and biological functions during DAI. The present study detected several novel biomarkers for noninvasively diagnosing and monitoring DAI and helped understand the DAI-associated metabolic events.

## 1. Introduction

Traumatic brain injury (TBI) represents a severe and complex worldwide and is associated with poor prognosis, long-term disabilities, and high mortality [1]. Diffuse axonal injury (DAI), with features of extensive axonal damage to the white matter of the brain, is more common in patients undergoing severe TBI [1, 2]. Recently, DAI has identified primary contributors to the clinical symptoms and poor functional and neurological prognosis of patients with TBI [3, 4]. The pathological mechanism of DAI is highly complicated and is still poorly understood [5]. Recently, axonal damage has been caused by direct shear or tensile forces of the brain and subsequent biochemical cascades postinjury [5]. The current gold standard for diagnosing DAI depends on a histopathological examination that reveals significant limitations in clinical application. Moreover, as axons are associated with the dissemination and microscopic characteristics, conventional neuroimaging approaches, including computed tomography (CT) or conventional magnetic resonance imaging (MRI), cannot visualize axonal histopathological changes due to head trauma, leading to a high rate of misdiagnosis [1]. Of note, an earlier identification of patients with DAI can influence physicians to determine appropriate therapeutic interventions to prevent axons from further damage, which may ultimately improve patient outcome. Thus, novel noninvasive diagnostic tools for DAI remain urgently within the neurological community to enable early diagnosis and appropriate therapeutic interventions.

In recent years, diffusion MRI has shown significant advantages in identifying pathological changes within the tissue microenvironment (below 100 mm) and could be used in the early diagnosis of DAI [1, 6]. Unfortunately, despite its higher adequate spatial sensitivity, the specificity and stability of the changes through diffusion MRI are inconsistent and insufficient for clinical implementation [1, 6]. Benjamini et al. used multidimensional MRI to identify biomarkers of axonal injury, and it may be a novel noninvasive method for detecting DAI [1]. However, this examination is costly and time-consuming, limiting the application scope. In addition to radiological procedures, omics-based research has also become an essential topic in developing noninvasive DAI biomarkers.

Urine and plasma are the most accessible source of biomarker analysis for numerous diseases, such as diabetes, acute kidney injury, and myocardial infarction. Our previous study conducted in the last few years has explored the metabolomic profile of DAI in plasma samples to gain a deeper understanding of the molecular mechanisms of DAI and identify novel and reliable biomarkers. Several plasma metabolites were identified as candidate biomarkers using an integrated ^1^H nuclear magnetic resonance spectroscopy (NMR) and ultraperformance liquid chromatography quadrupole-time-of-flight hybrid mass spectrometry- (UPLC/Q-TOF MS-) based metabolomics approach [7]. However, there still exist some limitations in the study, such as only one time point was concerned and the dynamic evolvement process of the identified metabolites was not studied. Moreover, urine presents unique advantages in the biomarker analysis, such as noninvasive, rapidly and easily obtained, and available in copious amounts [8]. Thus, urine is an optimal specimen to develop biomarkers to diagnose and monitor DAI survival noninvasively. Metabolomics, which focuses on providing an unbiased view of changes in endogenous metabolites, has been successfully applied for identifying diagnostic biomarkers of many diseases [9–12]. At present, one of the most powerful analytical technologies for nontargeted metabonomic mapping is ultraperformance liquid chromatography quadrupole-time-of-flight hybrid mass spectrometry (UPLC/Q-TOF MS) technology, which could accurately quantify and discover the remarkably altered metabolites in biofluids or tissues. Recently, UPLC/Q-TOF MS-based metabolomics approach has been widely used in the study of disease diagnosis and the associated mechanisms [13, 14].

Therefore, the current work focused on understanding the urine metabolome features and identifying differentially changed metabolites of DAI. A well-established rat model and the UPLC/Q-TOF MS-untargeted metabolomics method would be utilized. Histopathological examination, multivariate pattern recognition, pathway analysis, and evaluation of prognostic ability were also carefully investigated. Moreover, this work is the first to detect biomarkers for noninvasively and reliably diagnosing DAI in urine specimens through metabolomics analysis. Our results identified novel possible biomarkers for diagnosing and monitoring DAI cases, thereby decreasing the use of harmful diagnostic approaches and may shed more light on those mechanisms based on extensive axonal injury posttrauma.

## 2. Material and Methods

### 2.1. Chemicals and Drugs

This work obtained acetonitrile, formic acid, and high-performance LC (HPLC) grade methanol from Tedia (Fairfield, OH, USA). The remaining reagents were analytically pure.

### 2.2. Animals and Ethics Statement

This work obtained 46 eight-week-old male adult Sprague-Dawley (SD) rats (weight, 250-300 g) from the Laboratory Animal Center of Chongqing Medical University (Chongqing, China). In addition, the animals were raised in a standard laboratory environment. All animal treatments in the present work strictly followed the Guide for the Care and Use of Laboratory Animals. The animal experiments gained approval from the Ethics Committee of Hainan Medical University (HYLL-2021-303).

### 2.3. Models and Sampling

#### 2.3.1. DAI Rat Model Establishment

Based on our previous work, we established the DAI rat model [15]. Briefly, we randomized 30 rats to develop the DAI rat model. Death was reported in seven animals shortly after injury (mortality, 23.3%). In contrast, the rest survived after the coma period (5.24 ± 1.36 min). Then, we randomized 16 survivors in the injury group and assigned them to two subgroups of eight rats each: 1 d (*n* = 8) group and 3 d (*n* = 8) group. Additionally, without damage, 16 sham rats were enrolled in the control group for equal treatment before the injury, including scalp incision and anesthesia. These animals were also assigned to two subgroups of eight rats each: 1 d control group or 3 d control group with eight rats each. The animals were sacrificed at the respective time points.

#### 2.3.2. Urine Sample Collection and Preparation

Twenty-four-hour urine samples were collected at 1 d or 3 d postinjury by placing rats in individual metabolic cages, followed by 10 min centrifugation at 13,000 rpm and 4°C to remove the insoluble impurities [8]. Supernatants were then transferred and preserved under -80°C before analysis. The supernatants within every urine specimen were thawed at ambient temperature and then added with 2 mL methanol under 10 s shaking through the vortex mixer. Later, the sample was subjected to 15 min centrifugation at 13,000 rpm and 4°C. Supernatants were subsequently filtered with the 0.22 *μ*m mesh to conduct UPLC/Q-TOF MS-based metabolomics.

#### 2.3.3. Histopathological Examination of Brain Tissue

Each rat was intraperitoneally injected with 100 mg/kg sodium pentobarbital at 1 d or 3 d after injury. After achieving an appropriate anesthetic level, the animals were euthanized through decapitation. Then, the brain tissue was immediately dissected, followed by fixation with 10% paraformaldehyde (PFA) for at least 12 h. Then, the brain tissue was sliced into 4 *μ*m paraffin sections, followed by hematoxylin and eosin (HE) staining. Later, sections were stained using Bielschowsky silver for validation.

### 2.4. UPLC/Q-TOF MS Analysis

The Eksigent UPLC system (Shimadzu Corporation, Kyoto, Japan) was used by combining with the AB SCIEX Triple-TOF 5600 mass spectrometry (MS) (Massachusetts, USA) for UPLC/Q-TOF MS-based urine metabolomic analysis. Autosampler and column temperatures were set to 4 and 40°C, respectively, and the injection volume was 5 *μ*L. Moreover, detailed procedures for metabonomics analysis have been reported previously [8, 16].

This work made samples for quality control (QC) as a pooled mixture of an aliquot of supernatants (20 *μ*L) collected in diverse urine specimens to avoid intrabatch variability and to evaluate whether the UPLC/Q-TOF MS system was stable and repeatable [16, 17]. The relative standard deviation (RSD) was determined to ensure that our metabolomic analysis system was suitably stable.

### 2.5. Data Analyses

The current work transformed raw data from metabolomics analysis to mzXML files, alignment, deconvolution, and normalization using MarkerView v1.2.1 software (AB SCIEX, Massachusetts, USA) before the multivariate analysis. Afterward, multivariate analysis was performed on those normalized data through the SIMCA-P software (v14.0, Umetrics, Umeå, Sweden). Meanwhile, orthogonal partial least squares discriminate analysis (OPLS-DA) and principal component analysis (PCA) were carried out to visualize discrimination in DAI urine compared with control samples. Multivariate models were analyzed for validity and robustness using the following parameters: *R*^2^*X*, *Q*^2^, and *R*^2^*Y*. We estimated that the OPLS-DA model-generated variable importance of project (VIP) values statistically indicated the significance of differences in injury compared with the control groups. Differential variables were selected based on three limitations: (1) VIP value > 1, (2) fold change (FC) of injury compared with control groups > 1.5, and (3) *p* value < 0.05. Those potential metabolites were identified by querying the exact accurate *m*/*z*, MS/MS spectra, and retention time pairs in the online database, including the HMDB. MetaboAnalyst 4.0 platform analyzed receiver operating characteristic (ROC) curves and validated biomarkers to determine whether the identified significantly changed metabolites could be the novel biomarkers. The online approach MetaboAnalyst was utilized to analyze the metabolic pathways enriched by those metabolites with significant FC.

### 2.6. Statistical Analysis

Biological factors were compared based on significant differences using Student's *t*-test among injury compared with control groups. SPSS21.0 statistical software (IBM, Armonk, NY, USA) was employed to undergo statistical analysis. *p* < 0.05 (two-sided) stood for a significant difference.

## 3. Results

### 3.1. DAI Model Histopathological Confirmation

**Figure 1:**
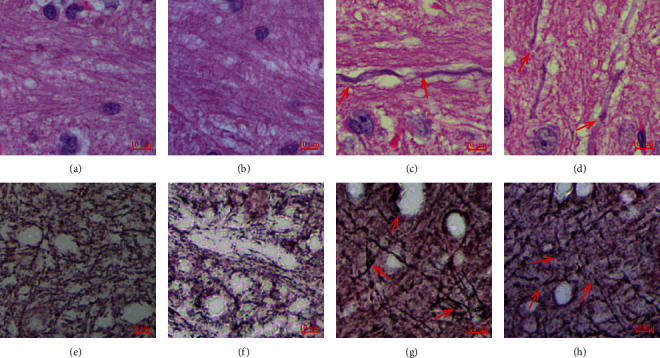
Brian tissues collected in injury and control groups were subjected to histopathological analysis. (a, e) 1 d control; (b, f) 3 d control; (c, g) 1 d; (d, h) 3 d groups. Tissues were examined after being stained with (a–d) HE and (e–h) Bielschowsky silver. This work collected each specimen in the corpus callosum. Axons of injury groups exhibited swellings and disconnection with ARBs to varying degrees (c, d, g, h; arrows), while the control group did not exhibit any abnormality in axons (a, b, e, f).

This work first conducted a histopathological examination of the brain tissue to detect axonal retraction balls (ARBs), disconnection, and axonal swelling to validate our constructed DAI rat models. ARBs could be seen within the affected rats' corpus callosum, which confirmed the successful establishment of the DAI rat model utilized in this work and its feasibility in subsequent UPLC/Q-TOF MS-based urine metabolomics. Representative histopathology sections of brain tissue are shown in Figure 1.

### 3.2. Urine Sample Metabolomics Data-Based Multivariate Analysis

**Figure 2:**
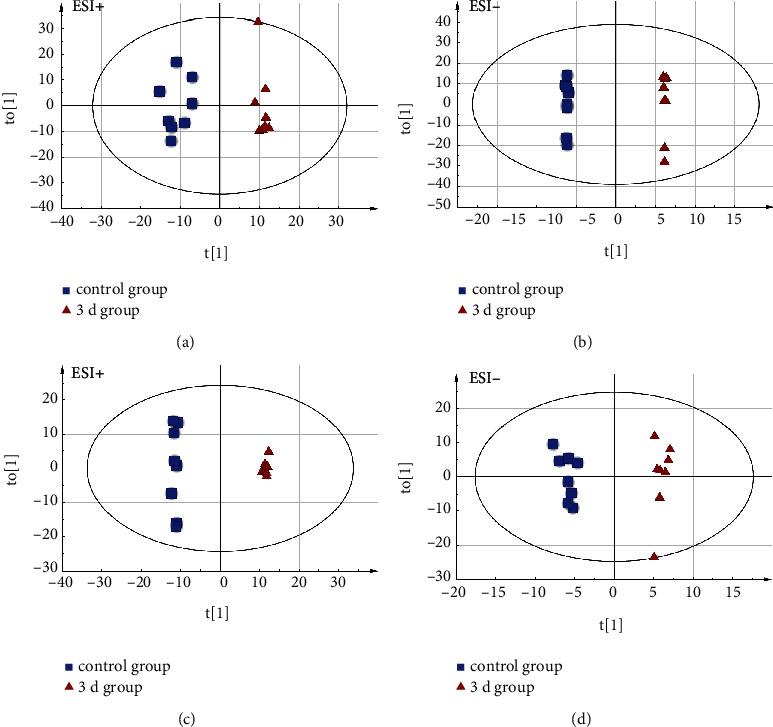
UPLC/Q-TOF MS-based OPLS-DA in the urine specimens collected from injury and control groups under positive and negative modes. (a, b) Score plots of the 1 d group were obtained by the UPLC/Q-TOF MS-based analyses under positive and negative modes. (c, d) Score plots of the 3 d group were obtained by the UPLC/Q-TOF MS-based analyses through positive and negative modes.

RSD values for QC samples established that our metabolomic analysis system was stable and suitable, indicating its feasibility in further analyses (Table S1). This work discovered 399 metabolites in urine samples, which were aligned, normalized, and utilized in multivariate regression. The PCA score plot revealed that the urine samples from the injured rats were distinct from controls, indicating the occurrence of urine metabolic disorder among DAI rats (Figure S1). OPLS-DA, a supervised clustering model, was undergone to elucidate differentially expressed factors of both groups. As depicted in the score plot, the 1 d group revealed clear segregation from control (Figures 2(a) and 2(b); ESI+: *R*^2^*X* = 0.491, *R*^2^*Y* = 0.99, and *Q*^2^ = 0.89; ESI-: *R*^2^*X* = 0.623, *R*^2^*Y* = 0.982, and *Q*^2^ = 0.942). It revealed the significant segregation of the control from the 3 d groups (Figures 2(c) and 2(d); ESI+: *R*^2^*X* = 0.573, *R*^2^*Y* = 0.946, and *Q*^2^ = 0.886; ESI-: *R*^2^*X* = 0.746, *R*^2^*Y* = 0.999, and *Q*^2^ = 0.944). Moreover, the DAI rats had robust metabolic alterations. Permutation tests (*n* = 300) were conducted to validate the OPLS-DA model. Therefore, our constructed OPLS-DA model revealed high validity and reliability (Figure S2, 1 d vs. the control group, ESI+: *R*^2^ = (0.0, 0.613), *Q*^2^ = (0.0, –0.123); ESI-: *R*^2^ = (0.0, 0.329), *Q*^2^ = (0.0, –0.071); Figure S3, 3 d vs. the control group, ESI+: *R*^2^ = (0.0, 0.735), *Q*^2^ = (0.0, –0.131); ESI-: *R*^2^ = (0.0, 0.465), *Q*^2^ = (0.0, –0.051)).

### 3.3. Differentially Expressed Metabolite Identification between the Plasma and Urine Specimens

**Figure 3:**
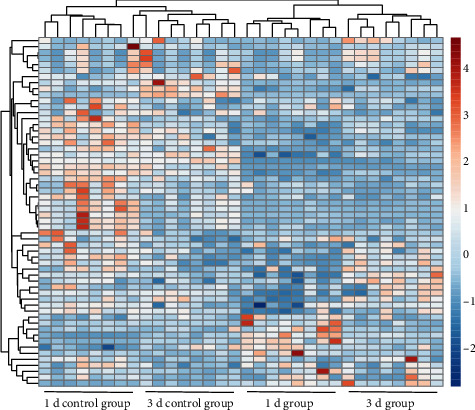
Heatmap depicting the hierarchical clustering analysis on injury and control urine samples. Red and blue stood for upregulated and downregulated metabolites separately. Injury groups were significantly different from the control group based on metabolites.

Depending on the S-plot from OPLS-DA, upon the thresholds of FC > 1.5 and VIP > 1, 30 factors between the 1 d group and control group were detected, including 13 under positive and 17 under negative modes (Figure S4A-S4B), as observed in Table S2. In the meantime, we discovered 30 metabolites with significant changes (21 under positive whereas nine in the negative modes, separately) from the 3 d group (Figure S4C-S4D; Table S3). There were 57 metabolites that had differential changes among injury groups by comparative analysis, among which three (urea, butyric acid, and taurine) showed significant differences between 1 d/3 d and control groups. Those 57 metabolites with significant changes were subject to clustering analysis to analyze the different metabolite signatures in injury compared with control groups. As a result, injury groups were separated from the control group, as observed in the heatmap (Figure 3).

ROC analysis was conducted to estimate whether the differential metabolites were the accurate biomarkers. Of those 57 metabolites, we identified seven metabolites showing area under the curve (AUC) > 0.9 as possible biomarkers in the 1 d group based on their high sensitivity and specialty (Table S4) and 13 metabolites in the 3 d group (Table S5). The AUC, together with the appropriate 95% confidence interval (CI) of the representative potential biomarker (taurine) in both 1 d and 3 d groups, was depicted in Figure S5.

### 3.4. Metabolic Pathway Analysis

**Figure 4:**
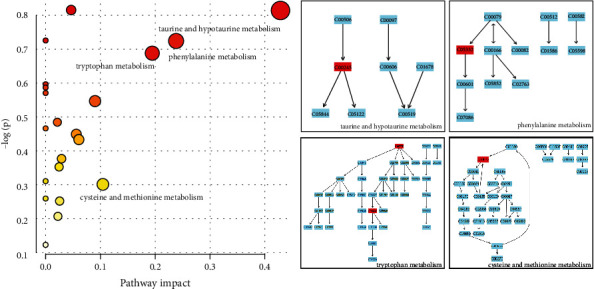
Those 57 metabolites enrich metabolic pathways with differential changes within the urine. The metabolic pathways were arranged by topology/enrichment analysis (*x*-/*y*-axes, separately), where *x*- and *y*-axes displayed pathway impact scores and *p* values separately. Circles have been colored according to specific *p* values, while circle size indicates the impact of the pathway.

Metabolites with significant changes were examined with MetaboAnalyst 4.0 to detect the most relevant pathways involved in the molecular mechanisms of DAI and to observe metabolic pathway changes comprehensively. As a result, 21 metabolic pathways showed the highest influence degrees following DAI (Figure 4). Four perturbed ones, including hypotaurine and taurine metabolism, phenylalanine metabolism, tryptophan metabolism, and cysteine and methionine metabolism, were selected because of high impact values related to the pathogenesis of DAI.

## 4. Discussion

The early diagnosis of DAI has a critical role in reducing mortality as it enables clinicians with robust evidence for determining appropriate therapeutic interventions during the early stages. While many studies have focused on identifying sensitive DAI biomarkers, none have been established [18]. Urine could be rapidly and easily obtained by the patients in a noninvasive manner. Moreover, the metabolite components and concentrations in urine are good indicators of metabolic fluctuations [8–12]. Thus, urinary metabolomic markers could collaborate to establish a more efficacious, cheap, and safe screening method for diagnosis of many diseases. Herein, we have addressed for the first time the analysis of metabolomic changes in urine of DAI. The UPLC/Q-TOF MS technology, combined with multivariate statistics analysis, has been applied in this study to obtain the urine metabolic profiles of rats with DAI. The primary objective of this study was to identify potential biomarkers in urine to improve early clinical diagnoses and monitor the prognosis of DAI.

In this study, urea was significantly upregulated in urine samples from injury groups compared to the control group. Urea is substantial nitrogen- (N-) containing end product of protein metabolism, having a critical effect on excreting dangerous N-containing substances [19, 20]. Ammonia will be transformed into urea during the urea cycle, while urea is later discharged through urine out of the body [19]. Previous studies showed that blood flow was decreased in the cerebral and energy metabolism was disturbed after TBI [15]. Glycolysis is enhanced while the tricarboxylic acid cycle (TAC) is inhibited after injury [21]. A study by Wang et al. indicated that amino acid-related metabolisms had been disturbed in acute ischemic stroke. The amino acids and urea were significantly downregulated in the serum [22]. In this work, an increase in urine urea is possibly associated with the dysfunction of amino acid metabolism postinjury which was consistent with our previous study [23]. Recent studies showed that glutamate was excess released following TBI, which induces metabolic energy failure and glutamate excitotoxicity [24]. Moreover, the excess released glutamate is reduced through the urea cycle and shortens the tricarboxylic acid cycle-induced glutamate oxidation [24]. Furthermore, previous research showed that brain urea played a pivotal role in neurodegenerative diseases and was significantly increased in Parkinson's disease dementia (PDD), Huntington's disease (HD), and Alzheimer's disease (AD) [20]. During DAI, the upregulated urea in urine could be an endogenous protective mechanism to reduce subsequent axonal damage postinjury. These findings indicate that clinical outcomes could be improved by promoting energy supply and urea excretion.

Butyric acid was also a selected metabolite biomarker, and it displayed significant downregulation among DAI urine samples than in controls. As a short-chain fatty acid, butyric acid is generated through colonic gut microbiota [25]. Recent studies have indicated that butyric acid participates in numerous biological processes (BPs), including thermogenesis, hemodynamics, inflammation, appetite, lipid/glucose metabolism, and gut microbial impact [25, 26]. Butyric acid produces a significant hypotensive and vasodilative effect as one of the mediators between gut microbiota and the circulatory system [25]. In addition, butyrate supplements are beneficial for metabolism, such as improving energy metabolism by decreasing energy absorption while increasing lipid oxidation, anti-intestinal inflammation, and regulating immunity [27, 28]. Previous studies also demonstrated that TBI had complicated activities in the gastrointestinal tract (GIT), and intestinal infection could worsen the brain lesion injury and negatively impact late outcomes among patients [29, 30]. Therefore, butyric acid possibly showed an essential role in the brain-gut axis, serving as the possible therapeutic target to reduce axotomy and improve outcomes among DAI patients.

In addition, another potential biomarker was taurine, which was involved in the remarkably perturbed metabolic pathways and taurine and hypotaurine metabolism. Taurine is a semiessential amino acid chiefly produced in the kidney and liver and present in various organs, such as the heart, brain, retina, muscle, placenta, and leukocytes [31, 32]. Taurine, the free amino acid with the highest abundance within the nervous system, has an essential nutritional effect on brain cell proliferation, development, and differentiation [32]. Previous studies have indicated that taurine administration could effectively mitigate neuronal damage severity and white matter injury in TBI and enhance cognitive impairment [33–35]. Recently, Daniel's group reported in lampreys that taurine enhances axon growth after complete spinal cord injury (SCI) [36]. Based on our results, taurine was downregulated in urine, possibly associated with an extracellular taurine increase in the central nervous system after injury and thus assisted with axotomy reduction and outcome improvement among DAI patients [37].

In research on DAI, our previous plasma proteomics and metabolomics study also found several significantly changed metabolites and proteins in rats with DAI [23]. Combining with results of this study, we found that significantly perturbed energy metabolism, inflammatory response, amino acid metabolism, cytoskeletal disruption, and immunomodulation may all participate in the axonal injury in DAI, which were consistent with previous studies [38, 39].

Above all, we built the DAI rat model and conducted the untargeted metabolomics study in urine samples to screen DAI-related urine metabolites having differential changes. Using the UPLC/Q-TOF MS-based metabonomics analysis, this work detected 57 metabolites with significant differences. Among them, we deemed three (urea, butyric acid, and taurine) possible diagnostic biomarkers which had essential effects on axonal damage during DAI. However, there are several limitations to our study. First, only one analytical platform was utilized, which could not obtain a holistic view of the molecular mechanisms of DAI. An integrated metabolomics and proteomics analysis may help to obtain a comprehensive picture of urine after DAI, and we would give more attention to this in our future studies. Second, the number of biological samples in our single-center pilot study was low. Hence, the results of this work should be validated, and efficient biomarkers to diagnose DAI should be identified through extensive investigations.

## Data Availability

The data used to support the findings of this study are available from the corresponding author upon request.

## Supplementary Materials

Supplementary Materials Table S1: the stability and reproducibility of the UPLC/Q-TOF MS analytical system were assessed by eight ions of the QC samples in both positive and negative ESI modes. Table S2: differentially changed metabolites in the urine samples of the 1 d group identified by UPLC/Q-TOF MS. Table S3: differentially changed metabolites in the urine samples of the 3 d group identified by UPLC/Q-TOF MS. Table S4: potential biomarkers in the urine samples of the 1 d group identified by UPLC/Q-TOF MS. Table S5: potential biomarkers in the urine samples of the 3 d group identified by UPLC/Q-TOF MS. Figure S1: score plots for PCA of UPLC/Q-TOF MS data from the control and injury groups. (A) ESI+, 1 d group; (B) ESI-, 1 d group; (C) ESI+, 3 d group; and (D) ESI-, 3 d group. Figure S2: the cross-validation plot of the OPLS-DA mode of UPLC/Q-TOF MS data from the control and 1 d group with 300 times permutation tests. (A) ESI+; (B) ESI-. Figure S3: cross-validation plot of the OPLS-DA mode of UPLC/Q-TOF MS data from the control and 3 d groups with 300 times permutation tests. (A) ESI+; (B) ESI-. Figure S4: S-plot from the OPLS-DA of UPLC/Q-TOF MS data in positive and negative ESI modes. (A, B) 1 d group; (C, D) 3 d group. Figure S5: ROC curve analysis was performed to evaluate the diagnostic accuracy of the selected metabolites. A representative potential biomarker, taurine, showed the highest predictive ability with AUC values of 0.9844.
