# Supplementary material for: UPLC/Q-TOF MS-Based Urine Metabonomics Study to Identify Diffuse Axonal Injury Biomarkers in Rat
Source: Dis Markers. 2022 Sep 21;2022:2579489. doi: 10.1155/2022/2579489 (PMC9519327; doi:10.1155/2022/2579489)
Supplement: Supplementary Materials — Table S1: the stability and reproducibility of the UPLC/Q-TOF MS analytical system were assessed by eight ions of the QC samples in both positive and negative ESI modes. Table S2: differentially changed metabolites in the urine samples of the 1 d group identified by UPLC/Q-TOF MS. Table S3: differentially changed metabolites in the urine samples of the 3 d group identified by UPLC/Q-TOF MS. Table S4: potential biomarkers in the urine samples of the 1 d group identified by UPLC/Q-TOF MS. Table S5: potential biomarkers in the urine samples of the 3 d group identified by UPLC/Q-TOF MS. Figure S1: score plots for PCA of UPLC/Q-TOF MS data from the control and injury groups. (A) ESI+, 1 d group; (B) ESI-, 1 d group; (C) ESI+, 3 d group; and (D) ESI-, 3 d group. Figure S2: the cross-validation plot of the OPLS-DA mode of UPLC/Q-TOF MS data from the control and 1 d group with 300 times permutation tests. (A) ESI+; (B) ESI-. Figure S3: cross-validation plot of the OPLS-DA mode of UPLC/Q-TOF MS data from the control and 3 d groups with 300 times permutation tests. (A) ESI+; (B) ESI-. Figure S4: S-plot from the OPLS-DA of UPLC/Q-TOF MS data in positive and negative ESI modes. (A, B) 1 d group; (C, D) 3 d group. Figure S5: ROC curve analysis was performed to evaluate the diagnostic accuracy of the selected metabolites. A representative potential biomarker, taurine, showed the highest predictive ability with AUC values of 0.9844. [file 2579489.f1.doc]

**Supplementary Material**

**Table of Contents**

1. Table S1. The stability and reproducibility of the UPLC/Q-TOF MS analytical system was assessed by eight ions of the QC samples in both positive and negative ESI mode. (Page S3)

2. Table S2. Differentially changed metabolites in the urine samples of 1 d group identified by UPLC/Q-TOF MS. (Page S4-S5)

3. Table S3. Differentially changed metabolites in the urine samples of 3 d group identified by UPLC/Q-TOF MS. (Page S6)

4. Table S4. Potential biomarkers in the urine samples of 1 d group identified by UPLC/Q-TOF MS. (Page S7)

5. Table S5. Potential biomarkers in the urine samples of 3 d group identified by UPLC/Q-TOF MS. (Page S8)

6.Fig. S1. Score plots for PCA analysis of UPLC/Q-TOF MS data from the control and injury groups. (A) ESI+, 1 d group; (B) ESI-, 1 d group; (C) ESI+, 3 d group; (D) ESI-, 3 d group. (Page S9)

7.Fig. S2. Cross-validation plot of OPLS-DA mode of UPLC/Q-TOF MS data from the control and 1 d group with 300 times permutation tests. (A) ESI+; (B) ESI-. (Page S10)

8.Fig. S3. Cross-validation plot of OPLS-DA mode of UPLC/Q-TOF MS data from the control and 3 d group with 300 times permutation tests. (A) ESI+; (B) ESI-. (Page S11)

9. Fig. S4. S-plot from the OPLS-DA analysis of UPLC/Q-TOF MS data in positive and negative ESI mode. (A-B) 1 d group; (C-D) 3 d group. (Page S12)

10. Fig. S5. ROC curve analysis was performed to evaluate the diagnostic accuracy of the selected metabolites. A representative potential biomarker, taurine, showed the highest predictive ability with AUC values of 0.9844. (Page S13)

Table S1. The stability and reproducibility of the UPLC/Q-TOF MS analytical system was assessed by eight ions of the QC samples in both positive and negative ESI mode.

| Peaks No. | ESI+ | | |  | ESI- | | |
| --- | --- | --- | --- | --- | --- | --- | --- |
|  | Retention time (min) | m/z | RSD (%) |  | Retention time (min) | m/z | RSD (%) |
| 1 | 3.46 | 214.1079 | 2.25 |  | 3.33 | 275.0241 | 3.24 |
| 2 | 13.39 | 118.0865 | 3.23 |  | 3.46 | 273.0087 | 2.59 |
| 3 | 2.10 | 150.0259 | 4.24 |  | 4.51 | 363.0184 | 5.12 |
| 4 | 12.80 | 101.0958 | 2.16 |  | 3.46 | 274.0113 | 4.28 |
| 5 | 4.19 | 302.1423 | 4.53 |  | 7.60 | 138.0202 | 1.24 |
| 6 | 14.30 | 87.0439 | 3.24 |  | 4.94 | 187.1346 | 5.04 |
| 7 | 2.01 | 126.0664 | 5.12 |  | 5.13 | 239.0943 | 3.25 |
| 8 | 6.87 | 205.1434 | 4.32 |  | 4.82 | 261.0449 | 4.14 |

Table S2. Differentially changed metabolites in the urine samples of 1 d group identified by UPLC/Q-TOF MS.

| No. | Metabolite | Retention time (min) | Ion (m/z) | VIP^a^ | P-value^b^ | Fold change^c^ | ESI mode |
| --- | --- | --- | --- | --- | --- | --- | --- |
| 1 | Dihydroferulic acid 4-O-sulfate | 3.33 | 275.0241 | 1.9 | 0.024 | -2.18 | ESI- |
| 2 | D-Glucuronic acid 1-phosphate | 3.46 | 273.0087 | 2.0 | 0.017 | -2.28 | ESI- |
| 3 | 4-hydroxy-5-[4-hydroxy-3-(sulfooxy)phenyl]pentanoic acid | 3.14 | 305.0345 | 2.0 | 0.013 | -3.11 | ESI- |
| 4 | Lorcaserin sulfamate | 3.46 | 274.0113 | 2.0 | 0.014 | -2.82 | ESI- |
| 5 | 6-Thioinosine-5'-monophosphate | 4.51 | 363.0184 | 2.1 | 0.009 | -2.34 | ESI- |
| 6 | 5'-(3',4'-Dihydroxyphenyl)-gamma-valerolactone sulfate | 3.43 | 286.9735 | 1.8 | 0.032 | -2.2 | ESI- |
| 7 | Oxalosuccinic acid | 2.35 | 188.9870 | 1.7 | 0.044 | 3.70 | ESI- |
| 8 | Secoisolariciresinol | 2.31 | 361.1502 | 1.8 | 0.042 | 2.54 | ESI- |
| 9 | Carbamic acid | 14.53 | 59.9878 | 3.4 | 0.000 | -28.70 | ESI- |
| 10 | Tiglylglycine | 2.81 | 156.0664 | 1.8 | 0.043 | -4.99 | ESI- |
| 11 | Pantoyllactone glucoside | 2.50 | 291.1082 | 2.0 | 0.023 | 2.16 | ESI- |
| 12 | Glucaric acid | 3.30 | 209.0302 | 1.8 | 0.049 | 2.07 | ESI- |
| 13 | Hydroxykynurenine | 2.84 | 223.0458 | 1.9 | 0.031 | 3.19 | ESI- |
| 14 | Ethylmalonic acid | 3.22 | 131.0349 | 2.4 | 0.006 | 2.26 | ESI- |
| 15 | Creatine riboside | 2.87 | 262.0925 | 1.9 | 0.03 | 2.33 | ESI- |
| 16 | Dihydrouracil | 2.67 | 113.0239 | 2.6 | 0.002 | 3.10 | ESI- |
| 17 | Aminomalonic acid | 4.05 | 118.0505 | 2.0 | 0.022 | 2.53 | ESI- |
| 18 | Urea | 14.90 | 61.0402 | 1.6 | 0.045 | 2.12 | ESI+ |
| 19 | 3-Hydroxyanthranilic acid | 3.46 | 214.1079 | 1.9 | 0.008 | -2.88 | ESI+ |
| 20 | Betaine | 13.39 | 118.0865 | 1.9 | 0.008 | -2.56 | ESI+ |
| 21 | Butyric acid | 4.68 | 89.0590 | 1.7 | 0.016 | -2.79 | ESI+ |
| 22 | 4-Methoxyphenylacetic acid | 13.72 | 173.0802 | 1.9 | 0.006 | -2.32 | ESI+ |
| 23 | 2-Octenedioic acid | 13.06 | 173.0809 | 1.6 | 0.027 | -2.44 | ESI+ |
| 24 | Phenylethylamine | 10.15 | 122.0966 | 2.2 | 0.001 | 2.47 | ESI+ |
| 25 | E-10-Hydroxynortriptyline | 13.49 | 280.1613 | 1.8 | 0.010 | -3.69 | ESI+ |
| 26 | (R)-2-Hydroxysterculic acid | 10.19 | 311.2550 | 2.1 | 0.002 | 1.2 | ESI+ |
| 27 | Taurine | 4.38 | 125.9863 | 1.3 | 0.02 | -2.49 | ESI+ |
| 28 | Acetic acid | 10.16 | 61.0285 | 1.4 | 0.034 | -1.47 | ESI+ |
| 29 | L-Methionine | 2.10 | 150.0259 | 1.6 | 0.029 | -1.69 | ESI+ |
| 30 | 3-Hexanone | 12.80 | 101.0958 | 1.4 | 0.000 | -3.13 | ESI+ |

^a^ VIP was obtained from OPLS-DA with a threshold of 1.0.

^b^ P-value were evaluated by two-tailed Student’s t-test.

^c^ Fold change (1 d/control) was calculated from the arithmetic mean values of two groups. The positive value indicate higher levels in 1 d group, and negative values indicate lower levels in 1 d group.

Table S3. Differentially changed metabolites in the urine samples of 3 d group identified by UPLC/Q-TOF MS.

| No. | Metabolite | Retention time (min) | Ion (m/z) | VIP^a^ | P-value^b^ | Fold change^c^ | ESI mode |
| --- | --- | --- | --- | --- | --- | --- | --- |
| 1 | 6-Hydroxynicotinic acid | 7.60 | 138.0202 | 2.1 | 0.008 | 2.22 | ESI- |
| 2 | 2-Hydroxydecanoate | 4.94 | 187.1346 | 1.9 | 0.015 | -4.03 | ESI- |
| 3 | 3-(3,4,5-Trimethoxyphenyl)propanoic acid | 5.13 | 239.0943 | 1.8 | 0.024 | -3.26 | ESI- |
| 4 | 5-Fluorouridine | 4.82 | 261.0449 | 2.0 | 0.010 | -2.67 | ESI- |
| 5 | Phenylacetylglutamine | 4.86 | 263.0603 | 1.9 | 0.017 | -2.67 | ESI- |
| 6 | 19-Noraldosterone | 2.48 | 345.1554 | 1.9 | 0.017 | -4.97 | ESI- |
| 7 | 3-Indole carboxylic acid glucuronide | 3.19 | 336.0726 | 1.8 | 0.049 | -4.49 | ESI- |
| 8 | 3-Methoxytyrosine | 5.15 | 210.0885 | 2.4 | 0.006 | 5.7 | ESI- |
| 9 | L-Arabinose | 2.12 | 149.0451 | 1.9 | 0.043 | 2.06 | ESI- |
| 10 | Butyric acid | 8.22 | 89.0590 | 1.8 | 0.006 | -2.00 | ESI+ |
| 11 | Cyclohexanone | 7.85 | 99.0801 | 2.3 | 0.000 | -2.00 | ESI+ |
| 12 | Oxymorphone | 4.19 | 302.1423 | 1.7 | 0.012 | -2.34 | ESI+ |
| 13 | Isocrotonic acid | 14.30 | 87.0439 | 1.6 | 0.023 | 2.00 | ESI+ |
| 14 | 3,7-Dimethyl-3-octene-1,2,6,7-tetrol | 5.07 | 205.1437 | 2.1 | 0.001 | -2.75 | ESI+ |
| 15 | Ethyl acetate | 13.25 | 89.0597 | 2.0 | 0.002 | -2.15 | ESI+ |
| 16 | Creatinine | 13.46 | 114.0910 | 1.4 | 0.043 | -2.42 | ESI+ |
| 17 | 2-Ethylacrylic acid | 4.48 | 101.0960 | 2.2 | 0.000 | -3.60 | ESI+ |
| 18 | 2-Pentylfuran | 2.06 | 139.1121 | 1.9 | 0.003 | -2.90 | ESI+ |
| 19 | LysoPA(P-16:0/0:0) | 11.88 | 395.2773 | 1.4 | 0.043 | -2.38 | ESI+ |
| 20 | Cervonoyl ethanolamide | 11.91 | 373.2945 | 1.4 | 0.043 | -2.90 | ESI+ |
| 21 | 2,6-Dimethylaniline | 4.26 | 122.0713 | 1.1 | 0.000 | -2.26 | ESI+ |
| 22 | Taurine | 4.38 | 125.9863 | 1.3 | 0.049 | -2.01 | ESI+ |
| 23 | 1-Methylhistamine | 2.01 | 126.0664 | 1.9 | 0.001 | 2.16 | ESI+ |
| 24 | L-Tryptophan | 6.87 | 205.1434 | 2.1 | 0.000 | -2.03 | ESI+ |
| 25 | gamma-Glutamylcysteine | 2.03 | 251.0369 | 1.8 | 0.003 | 4.18 | ESI+ |
| 26 | Stearidonyl carnitine | 4.89 | 420.3322 | 1.5 | 0.017 | -6.33 | ESI+ |
| 27 | Acetone | 9.87 | 59.0491 | 1.7 | 0.006 | 4.78 | ESI+ |
| 28 | Guanidine | 2.89 | 60.0449 | 1.5 | 0.02 | -3.22 | ESI+ |
| 29 | Urea | 3.54 | 61.0291 | 1.3 | 0.038 | 2.22 | ESI+ |
| 30 | Aminoacetone | 3.49 | 74.0602 | 1.6 | 0.008 | 6.75 | ESI+ |

^a^ VIP was obtained from OPLS-DA with a threshold of 1.0.

^b^ P-value were evaluated by two-tailed Student’s t-test.

^c^ Fold change (3 d/control) was calculated from the arithmetic mean values of two groups. The positive value indicate higher levels in 3 d group, and negative values indicate lower levels in 3 d group.

Table S4. Potential biomarkers in the urine samples of 1 d group identified by UPLC/Q-TOF MS.

| No. | Metabolite | Retention time (min) | Ion (m/z) | VIP^a^ | P-value^b^ | Fold change^c^ | ESI mode |
| --- | --- | --- | --- | --- | --- | --- | --- |
| 1 | Ethylmalonic acid | 3.22 | 131.0349 | 2.4 | 0.006 | 2.26 | ESI- |
| 2 | Dihydrouracil | 2.67 | 113.0239 | 2.6 | 0.002 | 3.10 | ESI- |
| 3 | Phenylethylamine | 10.15 | 122.0966 | 2.2 | 0.001 | 2.47 | ESI+ |
| 4 | Butyric acid | 4.68 | 89.0590 | 1.7 | 0.016 | -2.79 | ESI+ |
| 5 | Urea | 14.90 | 61.0402 | 1.6 | 0.045 | 2.12 | ESI+ |
| 6 | Taurine | 4.38 | 125.9863 | 1.3 | 0.02 | -2.49 | ESI+ |
| 7 | 3-Hexanone | 12.80 | 101.0958 | 1.4 | 0.000 | -3.13 | ESI+ |

^a^ VIP was obtained from OPLS-DA with a threshold of 1.0.

^b^ P-value were evaluated by two-tailed Student’s t-test.

^c^ Fold change (1 d/control) was calculated from the arithmetic mean values of two groups. The positive value indicate higher levels in1 d group, and negative values indicate lower levels in 1 d group.

Table S5. Potential biomarkers in the urine samples of 3 d group identified by UPLC/Q-TOF MS.

| No. | Metabolite | Retention time (min) | Ion (m/z) | VIP^a^ | P-value^b^ | Fold change^c^ | ESI mode |
| --- | --- | --- | --- | --- | --- | --- | --- |
| 1 | 2-Hydroxydecanoate | 4.94 | 187.1346 | 1.9 | 0.015 | -4.03 | ESI- |
| 2 | 3-Methoxytyrosine | 5.15 | 210.0885 | 2.4 | 0.006 | 5.7 | ESI- |
| 3 | Butyric acid | 8.22 | 89.0590 | 1.8 | 0.006 | -2.00 | ESI+ |
| 4 | Cyclohexanone | 7.85 | 99.0801 | 2.3 | 0.000 | -2.00 | ESI+ |
| 5 | Taurine | 4.38 | 125.9863 | 1.3 | 0.049 | -2.01 | ESI+ |
| 6 | 3,7-Dimethyl-3-octene-1,2,6,7-tetrol | 5.07 | 205.1437 | 2.1 | 0.001 | -2.75 | ESI+ |
| 7 | Ethyl acetate | 13.25 | 89.0597 | 2.0 | 0.002 | -2.15 | ESI+ |
| 8 | 2-Ethylacrylic acid | 4.48 | 101.0960 | 2.2 | 0.000 | -3.60 | ESI+ |
| 9 | Urea | 3.54 | 61.0291 | 1.3 | 0.038 | 2.22 | ESI+ |
| 10 | 1-Methylhistamine | 2.01 | 126.0664 | 1.9 | 0.001 | 2.16 | ESI+ |
| 11 | L-Tryptophan | 6.87 | 205.1434 | 2.1 | 0.000 | -2.03 | ESI+ |
| 12 | Acetone | 9.87 | 59.0491 | 1.7 | 0.006 | 4.78 | ESI+ |
| 13 | Aminoacetone | 3.49 | 74.0602 | 1.6 | 0.008 | 6.75 | ESI+ |

^a^ VIP was obtained from OPLS-DA with a threshold of 1.0.

^b^ P-value were evaluated by two-tailed Student’s t-test.

^c^ Fold change (3 d/control) was calculated from the arithmetic mean values of two groups. The positive value indicate higher levels in 3 d group, and negative values indicate lower levels in 3 d group.


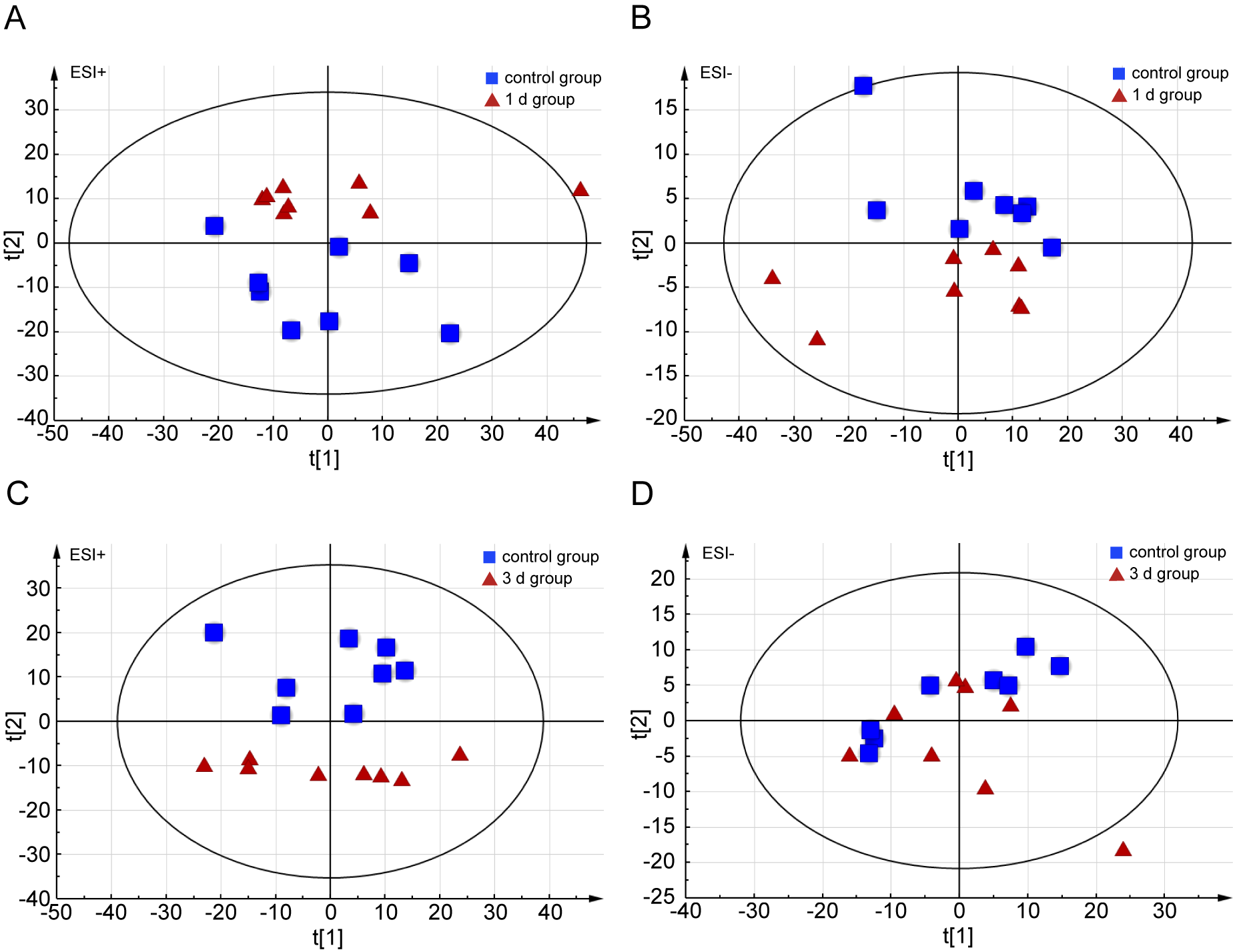


Fig. S1. Score plots for PCA analysis of UPLC/Q-TOF MS data from the control and injury groups. (A) ESI+, 1 d group; (B) ESI-, 1 d group; (C) ESI+, 3 d group; (D) ESI-, 3 d group.


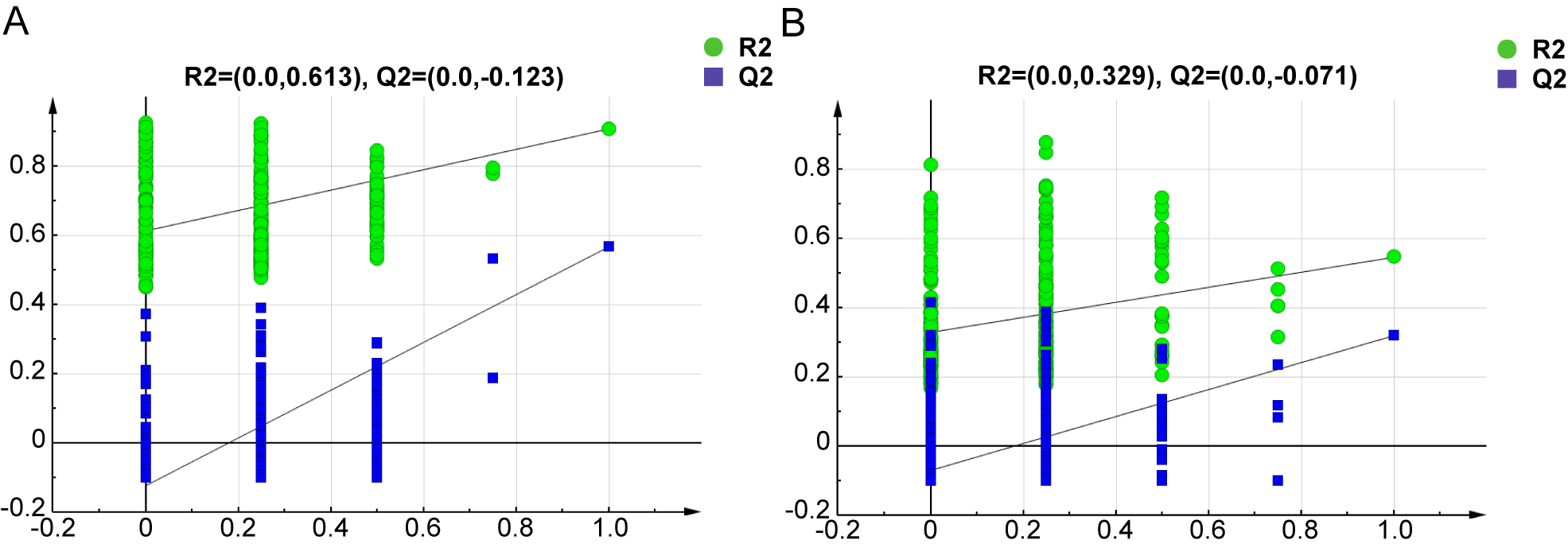


Fig. S2. Cross-validation plot of OPLS-DA mode of UPLC/Q-TOF MS data from the control and 1 d group with 300 times permutation tests. (A) ESI+; (B) ESI-.


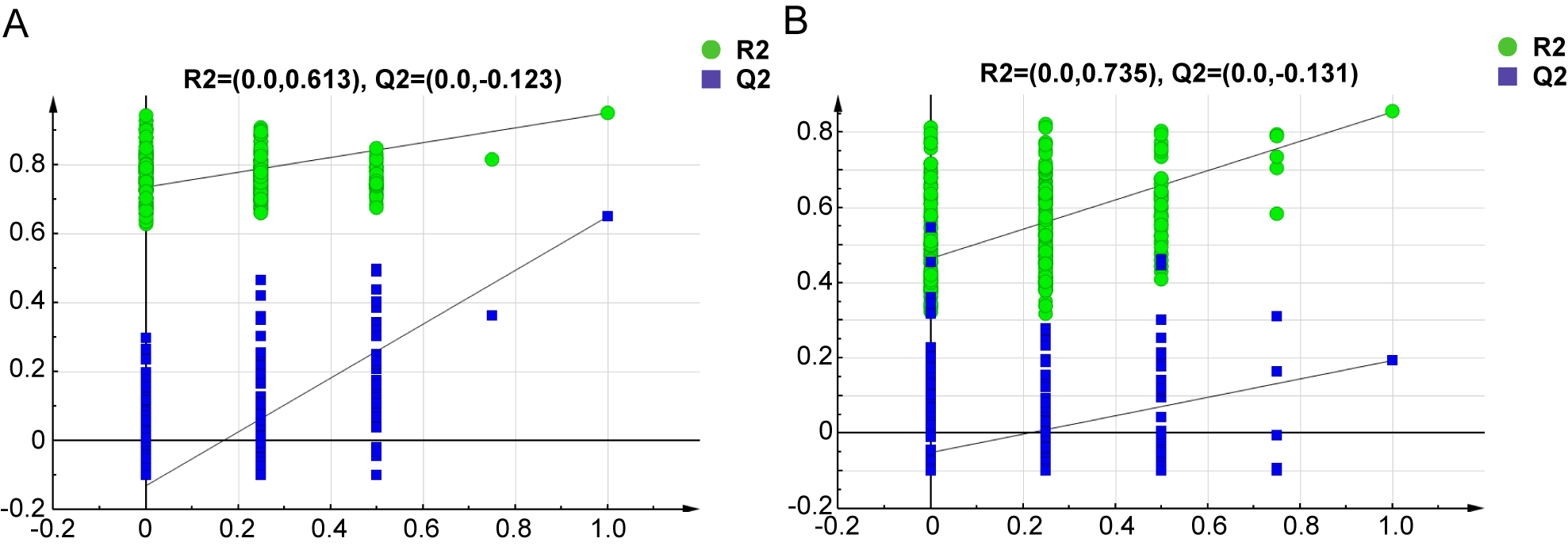


Fig S3. Cross-validation plot of OPLS-DA mode of UPLC/Q-TOF MS data from the control and high-dose group with 300 times permutation tests. (A) ESI+; (B) ESI-.


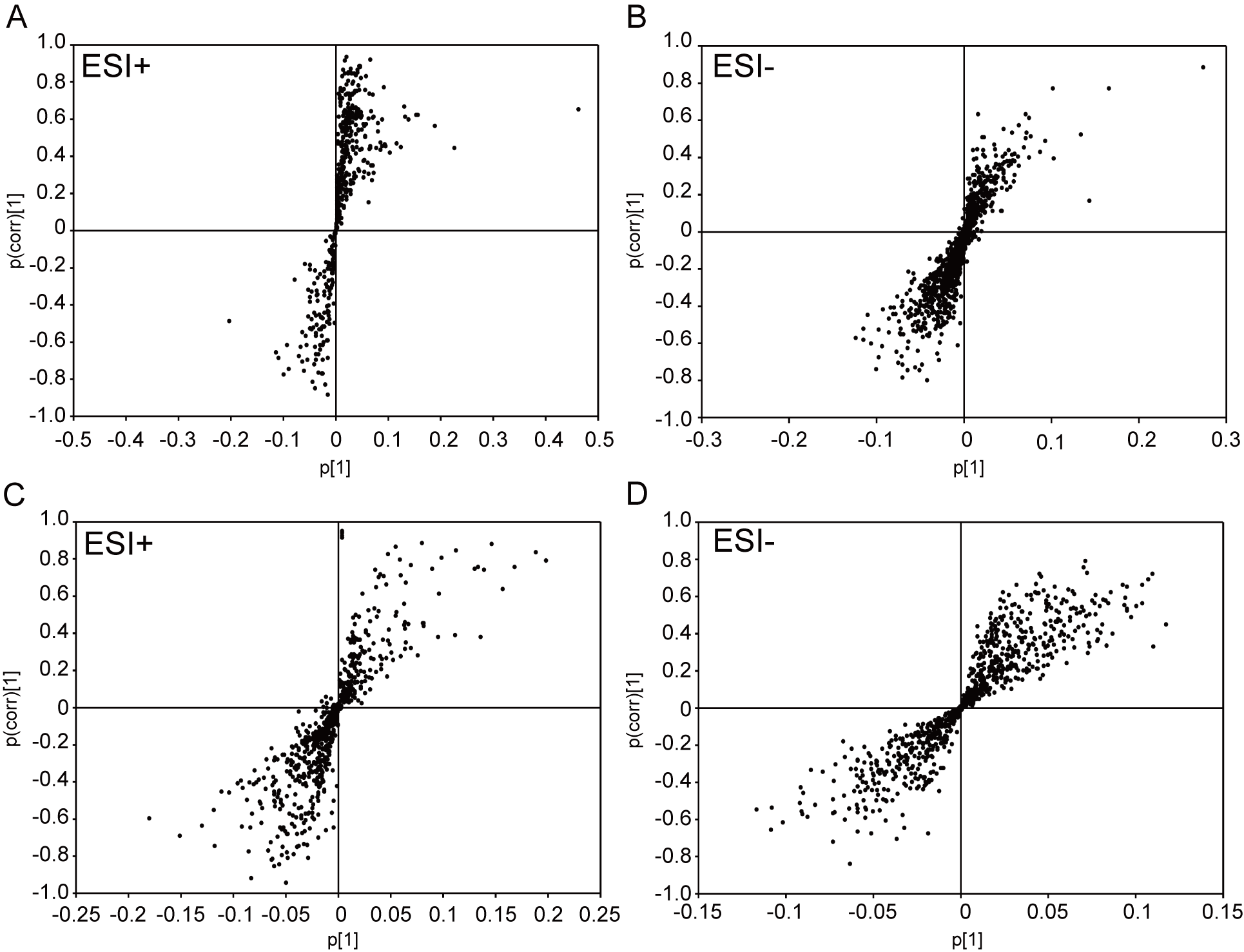


Fig. S4. S-plot from the OPLS-DA analysis of UPLC/Q-TOF MS data in positive and negative ESI mode. (A-B) 1 d group; (C-D) 3 d group.


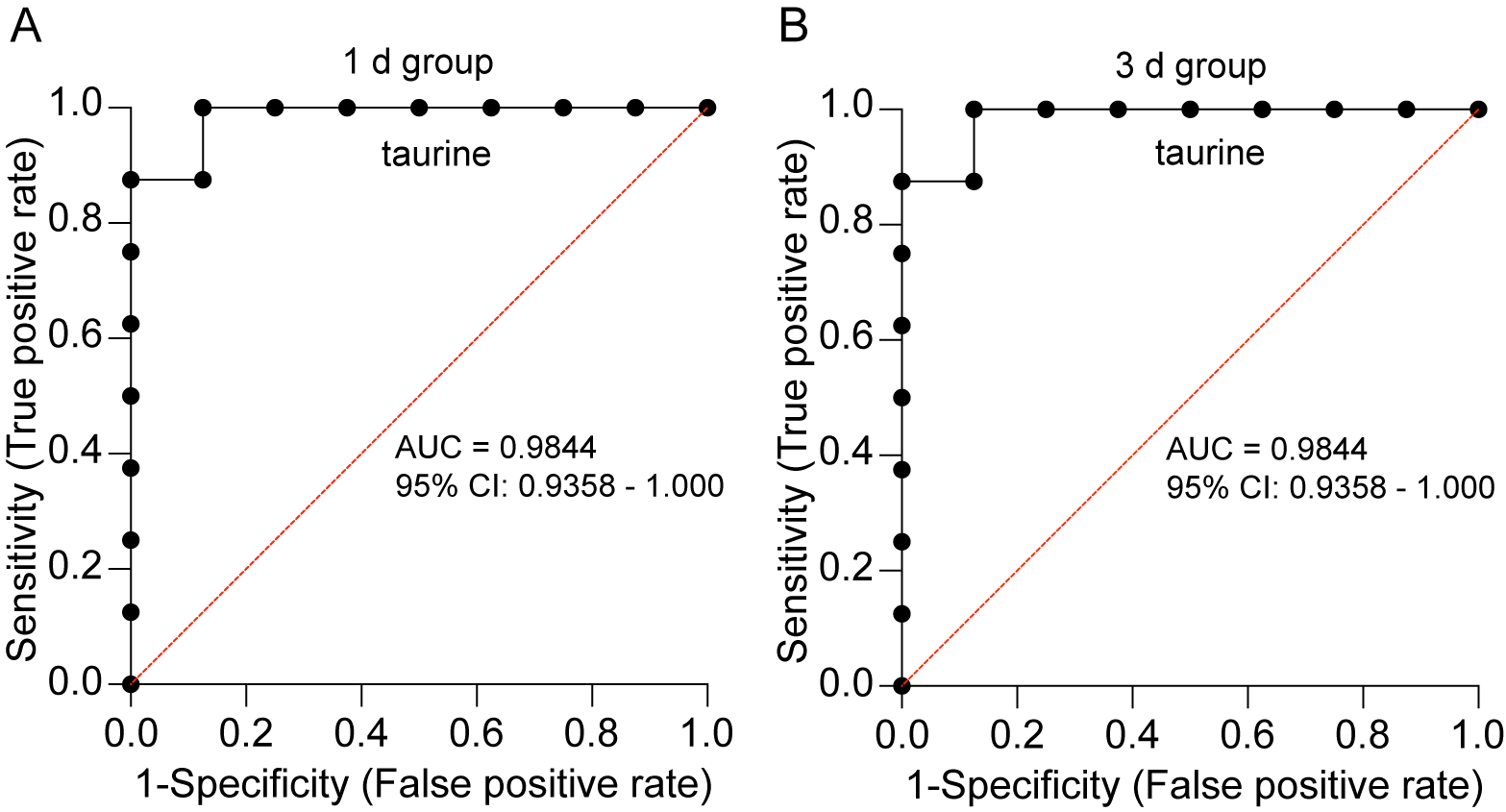


Fig. S5. ROC curve analysis was performed to evaluate the diagnostic accuracy of the selected metabolites. A representative potential biomarker, taurine, showed the highest predictive ability with AUC values of 0.9844.
